# Genetically-Determined Hyperfunction of the S100B/RAGE Axis Is a Risk Factor for Aspergillosis in Stem Cell Transplant Recipients

**DOI:** 10.1371/journal.pone.0027962

**Published:** 2011-11-17

**Authors:** Cristina Cunha, Gloria Giovannini, Antonio Pierini, Alain S. Bell, Guglielmo Sorci, Francesca Riuzzi, Rosario Donato, Fernando Rodrigues, Andrea Velardi, Franco Aversa, Luigina Romani, Agostinho Carvalho

**Affiliations:** 1 Microbiology Section, Department of Experimental Medicine and Biochemical Sciences, University of Perugia, Perugia, Italy; 2 Division of Hematology and Clinical Immunology, Department of Clinical and Experimental Medicine, University of Perugia, Perugia, Italy; 3 Anatomy Section, Department of Experimental Medicine and Biochemical Sciences, University of Perugia, Perugia, Italy; 4 Life and Health Sciences Research Institute, School of Health Sciences, University of Minho, Braga, Portugal; 5 Life and Health Sciences Research Institute/3B's - PT Government Associate Laboratory, Braga/Guimarães, Portugal; Universidade de Sao Paulo, Brazil

## Abstract

Invasive aspergillosis (IA) is a major threat to the successful outcome of hematopoietic stem cell transplantation (HSCT), although individual risk varies considerably. Recent evidence has established a pivotal role for a danger sensing mechanism implicating the S100B/receptor for advanced glycation end products (RAGE) axis in antifungal immunity. The association of selected genetic variants in the S100B/RAGE axis with susceptibility to IA was investigated in 223 consecutive patients undergoing HSCT. Furthermore, studies addressing the functional consequences of these variants were performed. Susceptibility to IA was significantly associated with two distinct polymorphisms in *RAGE* (-374T/A) and *S100B* (+427C/T) genes, the relative contribution of each depended on their presence in both transplantation counterparts [patient SNP*_RAGE_*, adjusted hazard ratio (HR), 1.97; *P* = 0.042 and donor SNP*_RAGE_*, HR, 2.03; *P* = 0.047] or in donors (SNP*_S100B_*, HR, 3.15; *P* = 7.8e-^4^) only, respectively. Functional assays demonstrated a gain-of-function phenotype of both variants, as shown by the enhanced expression of inflammatory cytokines in *RAGE* polymorphic cells and increased S100B secretion *in vitro* and *in vivo* in the presence of the *S100B* polymorphism. These findings point to a relevant role of the danger sensing signaling in human antifungal immunity and highlight a possible contribution of a genetically-determined hyperfunction of the S100B/RAGE axis to susceptibility to IA in the HSCT setting.

## Introduction

Invasive aspergillosis (IA) is a disease typically affecting high-risk patients such as that undergoing allogeneic hematopoietic stem cell transplantation (HSCT). Despite antifungal therapy, IA remains a leading cause of death among transplant recipients with a 1-year mortality reaching 75% [1]–[2]. Although displaying apparently similar “immunocompromised” phenotypes, not all HSCT recipients eventually develop disease, suggesting that genetically-determined immune defects may also play a role in defining susceptibility to IA [3]. In effect, single nucleotide polymorphisms (SNPs) in *TLR4* and *DECTIN1* have been recently proposed as strong predictive factors for incidence of IA [4]–[5] or fungal colonization [6] following HSCT.

Pathogen recognition through pathogen-associated molecular patterns (PAMPs) and from reaction to tissue damage-associated molecular patterns (DAMPs) is critical in establishing inflammation and resistance to infection [7]–[8]. However, unrestrained inflammation may also hamper the normal eradication of infection [9]. DAMPs such as high mobility group box 1 (HMGB1) and S100 proteins mediate inflammatory reactions through interaction with the multiligand receptor for advanced glycation end products (RAGE) [10]–[13]. Indeed, ligand-RAGE engagement has been highlighted as an amplifying mechanism of inflammation in immune/inflammatory diseases through a positive feedback loop between ligand availability and receptor expression [14].

Recently, a role for DAMP signaling has also been demonstrated in antifungal immunity [15]. S100B, a Ca_2_^+^-binding protein of the EF-hand type [16], was found to be critical for the integration of pathogen- and danger-sensing pathways to restrain inflammation in *Aspergillus fumigatus* infection [15]. However, when in excess, it adversely affected infection's outcome via the inflammatory RAGE pathway [15]. Based on this experimental evidence, polymorphisms within regulatory elements or ligand binding sites may potentially orchestrate RAGE's functional activity as well as ligand accumulation, so that certain individuals may be predisposed to heightened inflammatory responses and failure to control infections. Indeed, the nonsynonymous G82S polymorphism has been found to promote glycosylation and increased ligand affinity, upregulating intracellular signaling pathways linked with modulation of proinflammatory genes [17]–[18]. A gain-of-function effect was also demonstrated for the -374T/A polymorphism, resulting in enhanced transcriptional activity by impairing the binding of regulatory elements to the gene promoter [19]–[20]. Elevated serum concentrations of S100B, associated with certain immuno-mediated diseases [16], have been linked with genetic variants in the *S100B* gene [21].

We investigated the association of functional polymorphisms in the S100B/RAGE axis with susceptibility to IA in 223 consecutive HSCT recipients. Mechanistically, functional studies are presented supporting a genetically-determined hyperfunction of the S100B/RAGE axis as an additional risk factor for the development of IA in HSCT patients.

## Methods

### Patients

To assess the role of the DAMP system in aspergillosis, we analyzed a cohort of 223 consecutive patients with hematological malignancies who underwent allogeneic T-cell-depleted HSCT in Perugia between 2003 and 2010, and respective donors. Patient characteristics included patient age and gender, relation donor-patient gender, underlying disease and stage, transplant matching, CMV serology of donors and patients, conditioning regimen, graft-versus-host disease (GVHD) and antifungal prophylaxis (Table 1). Grafts consisted of immunoselected CD34^+^ peripheral blood cells in all cases and transplantation procedures were performed as described [22]. No GVHD prophylaxis or granulocyte colony-stimulating factor was administered after transplantation. Steroid therapy was performed in patients with diagnosed GVHD. Study approval was provided by the local ethics committee (Umbria Regional Hospital Ethics Committee, CEAS Umbria) and informed written consent was obtained from all participants in accordance with the Declaration of Helsinki.

**Table 1:** Patient, disease and transplantation characteristics (N = 192).

Characteristic	No IA (n = 151)	IA* (n = 41)	*P* †
**Age at transplantation, years [median (range)]**	38 (6–68)	39 (14–66)	0.65
**Sex, no. (%) male**	73 (48)	18 (44)	0.65
**Sex of donor/patient pair**			
Female/male	37 (25)	7 (17)	
Others	114 (75)	34 (83)	0.31
**HLA matching, no. (%)**			
HLA-identical sibling	64 (42)	11 (27)	
One HLA haplotype-mismatched family member	87 (58)	30 (73)	0.05
**Underlying disease, no. (%)**			
Acute leukemia	105 (69)	23 (56)	
Lymphoma/myeloma	36 (24)	13 (32)	
Chronic leukemia	10 (7)	5 (12)	0.31
**Advanced disease stage, no. (%)**	98 (65)	30 (73)	0.29
**Conditioning regimen, no. (%)**			
With TBI	108 (72)	34 (83)	
Without TBI	43 (28)	7 (17)	0.16
**CMV serology of donor and recipient, no. (%)**			
CMV^-^/CMV^-^	15 (10)	4 (10)	
CMV^-^/CMV^+^, CMV^+^/CMV^-^ or CMV^+^/CMV^+^	136 (90)	37 (90)	0.94
**GVHD, grade II to IV, no. (%)**	5 (3)	5 (12)	0.04
**Antifungal prophylaxis, no (%)**			
Liposomal amphotericin-B	127 (84)	40 (98)	
Fluconazole	23 (16)	1 (2)	0.03

IA – invasive aspergillosis; HLA – human leukocyte antigen; TBI – total body irradiation; CMV – cytomegalovirus; GVHD – graft-versus-host-disease.

*Patients diagnosed with possible aspergillosis (n = 31) were excluded from the study.

† *P* values are from Gray's test using cumulative incidence analysis.

Antifungal prophylaxis included liposomal amphotericin-B (1 mg/kg daily) in high-risk patients (n = 195; 87.4%) and fluconazole (400 mg daily) in standard risk patients (n = 28; 12.6%) from day −5 until neutropenia ended. Criteria defining standard and high risk for IA were applied as described [23]. Surveillance cultures from stool, urine, nasal and oral washes were performed at the time of transplantation. Additionally, blood, sputum, bronchoalveolar lavages (BAL), serum galactomannan (Platelia *Aspergillus* EIA, Bio-Rad, Hercules, CA) and cultures of samples were analyzed when clinical symptoms of infection appeared. Probable/proven fungal infection was defined according to the revised standard criteria from the European Organization for Research and Treatment of Cancer/Mycology Study Group (EORTC/MSG) [24].

### DNA isolation and SNP genotyping

Genomic DNA from patients and donors was isolated from whole blood before transplantation using the QIAamp DNA Blood Mini kit (Qiagen, Milan, Italy) according to manufacturer's instructions. The *RAGE* (rs2070600, −374T/A and rs1800624, G82S) and *S100B* (rs9722, +427C/T) polymorphisms were selected from a literature review and public databases based on three selection criteria: i) published evidence of association with human diseases [18]–[21], ii) localization to the promoter, untranslated (UTR) or coding sequence, and (iii) minor allele frequencies higher than 5% in the Caucasian population. Genotyping was performed using bi-directional PCR amplification of specific alleles (Bi-PASA) as previously described [25], or using allele-specific PCR. Primer sequences are described in Table S1. Genotyping was validated by direct sequencing of at least 30 randomly selected DNA samples from either patients or donors for each polymorphism. Genotyping sets comprised randomly selected replicates of previously typed samples and two negative controls (water). Concordant genotyping was obtained for ≥99% assays.

### *In silico* miRNA prediction

The +427C/T polymorphism within the 3′-UTR region of the *S100B* gene was analyzed in order to predict and compare alteration of potential microRNA targeting sites. Analysis was performed using the TargetScan version 4.0 (http://www.targetscan.org/) and microSNiPer (http://cbdb.nimh.nih.gov/microsniper) public databases. A minimum “seed” length of 7-mer was specified as minimum cut-off score.

### Reagents

Recombinant bovine S100B, 96% identical to human S100B, was expressed and purified as reported [26]–[27]. Purified S100B was passed through an END–X B15 Endotoxin Affinity Resin column (Associates of Cape Cod, Inc., East Falmouth, MA) to remove contaminating bacterial endotoxin. Endotoxin levels (<1.0 EU/mL) were verified by a standard assay using a *Limulus* amoebocyte lysate reagent test kit (Associates of Cape Cod, Inc.). S100B concentration was calculated using the molecular weight of the S100B dimer (21 kDa). Recombinant HMGB1 was a kind gift from Heikki Rauvala (University of Helsinki, Helsinki, Finland).

### Cell preparation, cultures and treatments

In order to assess the functional consequences of the genetic variants in the S100B/RAGE axis, we resorted to human cells from individuals bearing distinct genotypes. Upon written informed consent, human peripheral blood mononuclear cells (PBMCs) from whole blood of healthy volunteers were isolated using sterile-filtered Histopaque®-1077 (Sigma-Aldrich, St. Louis, MO) according to manufacturer's instructions. Cells (2.5×10^6^ cells/mL) were stimulated with S100B (4 nM and 4 µM), HMGB1 (300 nM), Zymosan (Zym, 10 µg/mL) or inactivated *A. fumigatus* conidia (1∶1 ratio) for 2 hours before RNA extraction or 12 hours before supernatant collection and cell lysis. The nanomolar versus micromolar concentrations of S100B were selected based on their antagonizing effects in inflammation, as previously described [15]. For each assay, cells from either wild-type (n = 10) or polymorphic (n = 10) individuals for the *RAGE* and *S100B* variants were used. Cells from the same individuals were used across conditions in each assay.

### BAL collection

To assess potential genetically-determined differences in S100B levels in IA patients, we measured S100B concentrations in BAL samples from HSCT recipients. Sample collection was performed as per EORTC/MSG criteria [24] in line with the respective institutional procedures and standardized according to the guidelines proposed by the European Respiratory Society to measure acellular components [28]. All patients were non-smokers without any other relevant associated disease (e.g. asthma) and were undergoing identical drug regimens. All BAL samples were obtained by the instillation of 150 ml of fluid and comparable recovery rate, therefore preventing errors associated with dilution and stored at -80°C until use.

### Real–time RT-PCR analysis

Real–time RT–PCR was performed using the iCycler iQdetection system (Bio–Rad) and SYBR Green chemistry (Finnzymes Oy, Espoo, Finland). Total RNA was extracted using RNeasy Mini Kit (QIAGEN) and was reverse transcribed with Sensiscript Reverse Transcriptase (QIAGEN) according to manufacturer's instructions. PCR primers are described in Table S1. Amplification efficiencies were validated and normalized against β-actin (*ACTB*). The thermal profile for real-time RT-PCR was 95°C for 3 min, followed by 40 cycles of denaturation for 30 s at 95°C and an annealing/extension step of 30 sec at 60°C. Each data point was examined for integrity by analysis of the amplification plot. mRNA–normalized data were expressed as relative mRNA expression in stimulated cells compared to that of wild-type untreated cells.

### ELISA assays

Supernatants from PBMC cultures were collected after stimulation and BAL samples from patients with IA were obtained before initiation of treatment. ELISA for S100B was performed as follows: 50 µl of sample were incubated overnight on a microtiter plate previously coated with a monoclonal mouse anti-S100B antibody (Sigma-Aldrich). A rabbit anti-S100B antibody (EPOS) conjugated with peroxidase (Dako, Copenhagen, Denmark) was then added for 1 hour before the color reaction with SIGMA*FAST*™ OPD peroxidase substrate (Sigma-Aldrich) was measured at 492 nm. The standard S100B curve ranged from 0.2 to 40 ng/mL.

### Western blotting

Blots of cell lysates were incubated with a goat polyclonal anti-RAGE IgG antibody (Santa Cruz Biotechnology, Santa Cruz, CA) followed by addition of horseradish peroxidase-conjugated anti-goat IgG secondary antibody (Cell Signaling Technology, Danvers, MA). Blots were developed with the Enhanced Chemiluminescence detection kit (Amersham Pharmacia Biotech, Milan, Italy). Control experiments included staining without the primary antibody.

### Statistical analysis

Consistency of genotype frequencies with the Hardy–Weinberg equilibrium was tested using a χ2 test on a contingency table of observed vs. predicted genotype frequencies (*P*>0.05). Probability of IA was determined using the Gray's test and analyzed using the cumulative incidence method. Cumulative incidences were computed with the *cmprsk* package for R 2.10.1 software [29], with the competing events for IA being relapse and death. Overall survival was defined as the time from first day of treatment to death from any cause and was obtained by the Kaplan-Meier method and compared using the log-rank test. Clinical co-variables depicted in Table 1, as well as donor and patient genotype, were tested in univariate analyses. GVHD was considered in the univariate analysis as a time-dependent covariate for IA. Variables with a *P* value ≤0.15, namely transplant matching, GVHD and antifungal prophylaxis, were included in the multivariate model. Multivariate analysis was performed for each polymorphism separately using the subdistribution regression model of Fine and Gray with the *cmprsk* package for R 2.10.1 software [30]. Functional data were analyzed by GraphPad Prism 4.03 program (GraphPad Software, San Diego, CA). Unpaired Student's *t*-test with Bonferroni's adjustment and 2-tailed Mann-Whitney rank-sum test were used to determine statistical significance (*P*<0.05).

## Results

### Polymorphisms in the S100B/RAGE axis and risk of IA in HSCT recipients

The G82S polymorphism in *RAGE* was eventually excluded from the analyses given it displayed a minor allele frequency lower than 5% in our patient cohort (data not shown). Genotype frequencies of the remaining *RAGE* and *S100B* polymorphisms were comparable among patients, donors and healthy individuals (Table 2) and were not significantly different with regard to the underlying hematological disease (Table S2) or antifungal prophylaxis (Table S3). Among the 223 patients enrolled in our study, 41 (18%) developed proven/probable IA within a median time of 97 days (range, 1–390 days) following HSCT. Thirty-four (15%) patients with diagnosis of possible infection or with history of pretransplantation disease were excluded from further analyses. Ninety-five (43%) patients were long-term survivors following transplantation; median follow-up time among surviving patients was 45 months (range, 5 to 100) and among those who died was 7 months (range, 1 to 57). No statistically significant influence of *RAGE* or *S100B* polymorphisms on patient survival was found (data not shown).

**Table 2:** Genotype distributions of *RAGE* and *S100B* polymorphisms in donors and recipients of stem cell transplants and healthy controls.

Genotype	Donor (n = 223)	Recipient (n = 223)	Healthy controls (n = 468)	*P* *
WT*_RAGE_*	108 (48.4%)	97 (43.5%)	215 (45.9%)	
SNP*_RAGE_*	115 (51.6%)	126 (56.5%)	253 (54.1%)	0.58
WT*_S100B_*	188 (84.3%)	182 (81.6%)	390 (83..3%)	
SNP*_S100B_*	35 (15.7%)	41 (18.4%)	78 (16.7%)	0.74

WT – wild-type; SNP – single nucleotide polymorphism. WT*_RAGE_* – TT genotype; SNP*_RAGE_* – TA + AA genotypes; WT*_S100B_* – CC genotype; SNP*_S100B_* – CT + TT genotypes.

*P values are calculated with the Freeman-Halton extension of the Fisher's exact test for a two-rows by three-columns contingency table.

**Figure 1:**
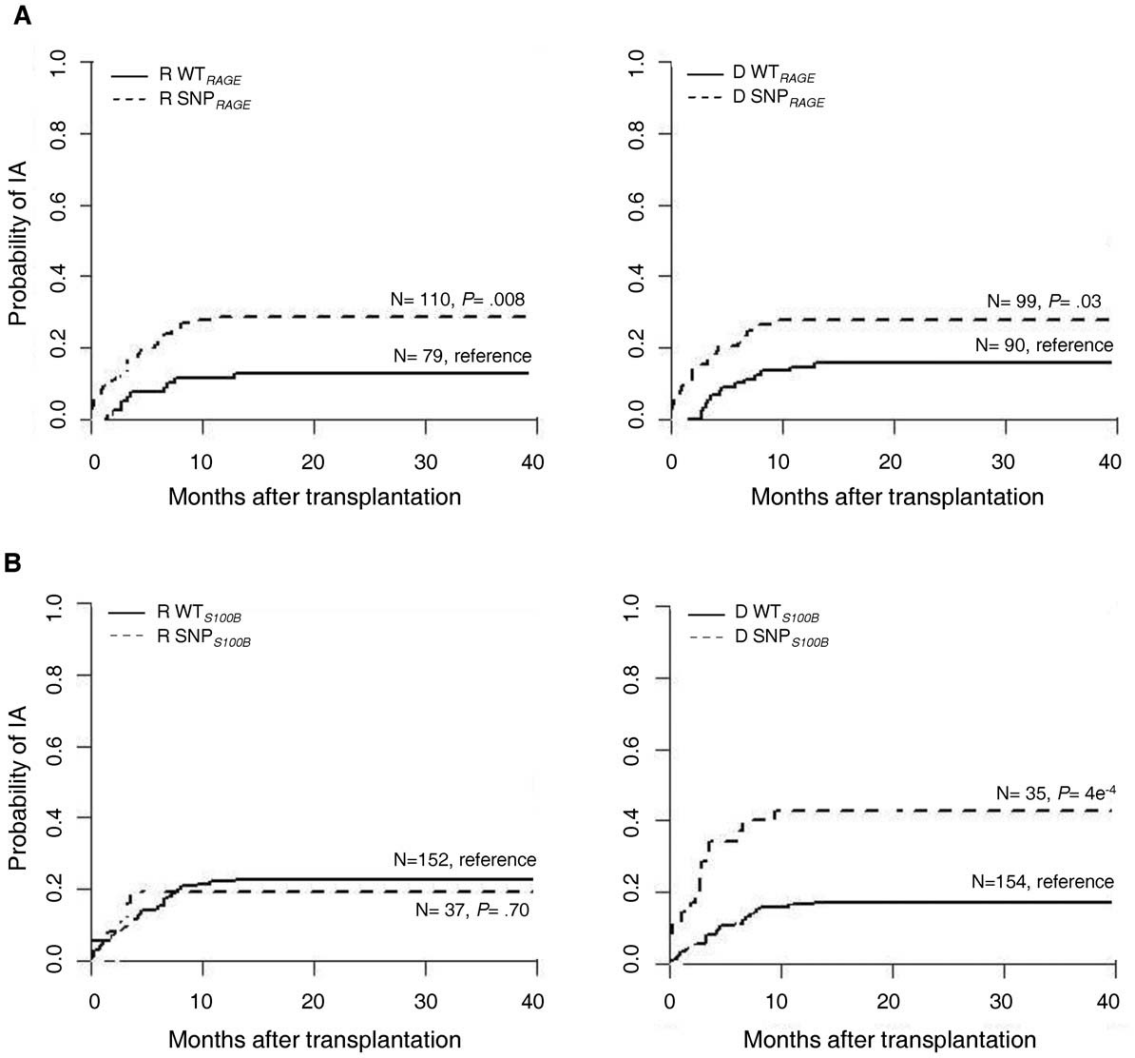
Polymorphisms in the S100B/RAGE axis and risk of IA in HSCT recipients. (*A*) Cumulative incidence of IA according to *RAGE* -374T/A genotype (WT*_RAGE_*, TT; SNP*_RAGE_*, TA+AA). (*B*) Cumulative incidence of IA according to *S100B* +427C/T genotype (CC, WT*_S100B_*; CT+TT, SNP*_S100B_*). From left to right, patients (R) and donors (D).

To estimate the risk of IA according to patient or donor genotypes, we determined cumulative incidences of IA among transplant recipients at the date of the last documented case. *RAGE* -374T/A polymorphism increased susceptibility to IA when present in either patients [12.7% WT*_RAGE_* (wild-type, TT genotype) *vs*. 28.6% SNP*_RAGE_* (TA+AA genotypes); *P* = 0.008] or donors (15.6% WT*_RAGE_ vs*. 27.7% SNP*_RAGE_*; *P* = 0.029) (Figure 1*A*). In contrast, *S100B* +427C/T polymorphism predisposed to IA when present in donors [17.2% WT*_S100B_* (wild-type, CC genotype) *vs*. 42.9% SNP*_S100B_* (CT+TT genotypes); *P* = 4e^−4^], but not in patients (22.7% WT*_S100B_ vs*. 18.9% SNP*_S100B_*; *P* = 0.70) (Figure 1*B*).

In the multivariate analysis, all genetic associations remained significant, as well as the clinical co-variables HLA-haplotype mismatching and GVHD (Table 3). SNP*_RAGE_* genotypes in patients and donors independently resulted in a 2-fold increased risk of IA after HSCT, when compared to their wild-type counterparts. Instead, donor SNP*_S100B_* genotype promoted a 3-fold increased risk of IA compared to WT*_S100B_* donors. It is also interesting to note that the effects of these polymorphisms were restricted to IA. In fact, no other clinical outcomes assessed in our cohort such as relapse, transplant-related mortality or GVHD, as well as infectious outcomes, specifically cytomegalovirus (CMV) disease, were influenced by *RAGE* or *S100B* polymorphisms (Figure S1).

**Table 3:** Multivariate analysis of the association of *RAGE* -374T/A and *S100B* +427C/T polymorphisms with risk of IA following HSCT.

Risk factors*	Adjusted HR	95% CI	*P*
*RAGE* -374T/A			
R SNP*_RAGE_*	2.03	1.01–4.10	0.047
D SNP*_RAGE_*	1.97	1.02–3.79	0.042
HLA mismatching	2.16	1.08–4.31	0.029
GVHD	3.21	1.26–8.20	0.015
*S100B* +427C/T			
D SNP*_S100B_*	3.15	1.61–6.15	7.8e^4^
HLA mismatching	1.99	1.03–3.87	0.042
GVHD	2.80	1.00–8.48	0.050

R: recipient; D: donor; HR: hazard ratio; CI: confidence interval; HLA: human leukocyte antigen; GVHD: graft-versus-host-disease; SNP*_RAGE_*, TA + AA genotypes; SNP*_S100B_*, CT + TT genotypes.

*For the genetic factors, R WT*_RAGE_*, D WT*_RAGE_* and D WT*_S100B_* genotypes are the referent categories, respectively.

### Functional consequences of *RAGE* and *S100B* polymorphisms

**Figure 2:**
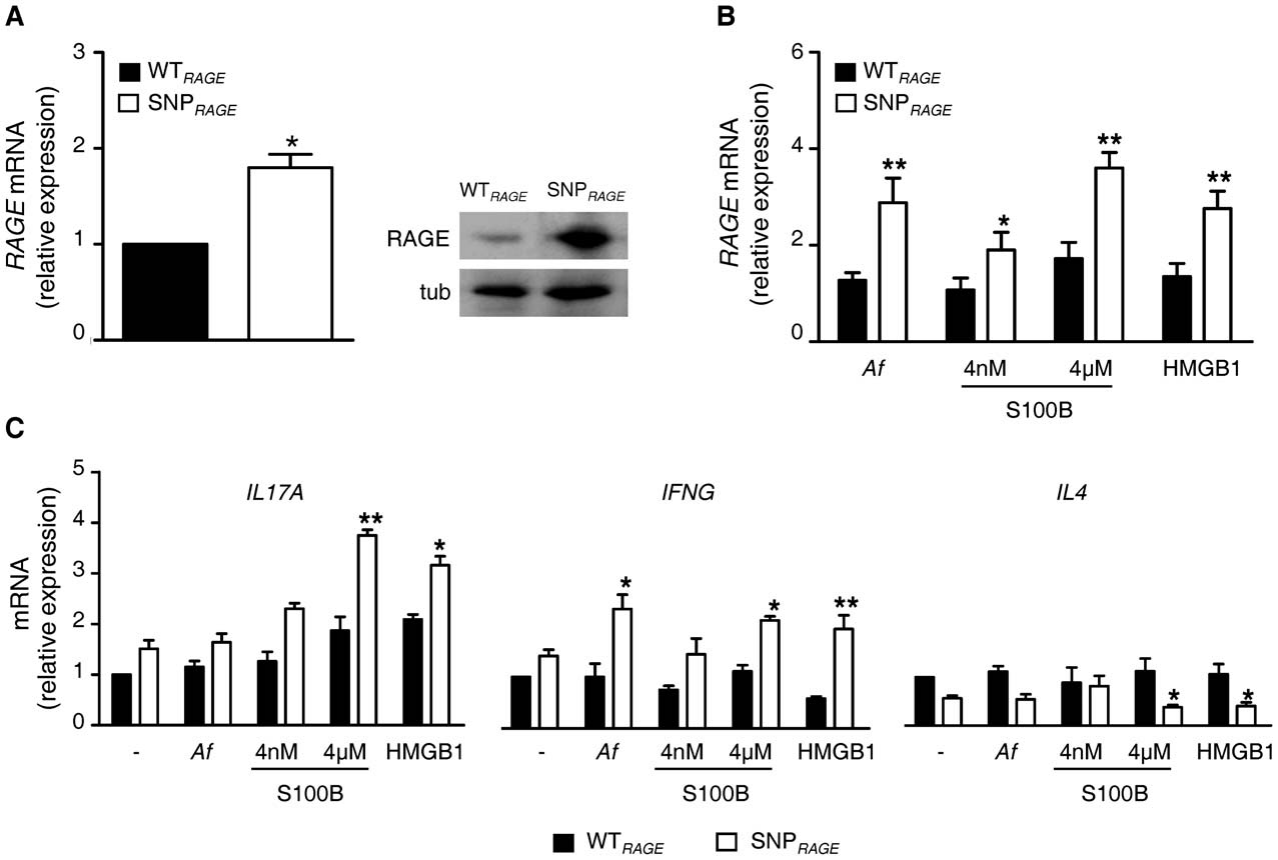
Functional consequences of *RAGE* -374T/A polymorphism. Analysis of RAGE expression in PBMCs from wild-type (WT*_RAGE_*, TT genotype) or mutant homozygous (SNP*_RAGE_*, AA genotype) individuals (*A*) untreated or (*B*) stimulated with *A. fumigatus* conidia (*Af*), S100B and HMGB1. In (*A*), a representative Western blot of RAGE in untreated WT*_RAGE_* and SNP*_RAGE_* PBMCs is shown. Tub, tubulin. (*C*) Cytokine gene expression in human PBMCs isolated from WT*_RAGE_* or SNP*_RAGE_* individuals. Data are shown as mRNA relative expression using untreated WT*_RAGE_* cells as reference (for every assay, shown is the mean±SD of data obtained for 10 individuals of each genotype; *P≤0.05 and **P≤0.01 by unpaired *t*-test). Cells from the same individuals were used across conditions in each assay.

To determine whether RAGE expression was altered by the -374T/A polymorphism, we analyzed WT*_RAGE_* (TT genotype) and SNP*_RAGE_* (AA genotype) PBMCs isolated from healthy individuals. Cells were either left untreated or stimulated with *A. fumigatus* conidia, S100B or HMGB1. We found that PBMCs carrying the SNP*_RAGE_* genotype displayed an increased expression of *RAGE* compared to WT*_RAGE_* cells (Figure 2*A*). Stimulation with *A. fumigatus* conidia, S100B (particularly at the micromolar dose) and HMGB1 further amplified this effect (Figure 2*B*). The fact that *RAGE* expression was modulated in cells pulsed with the fungus confirms the important role of the RAGE pathway in antifungal immunity [15].

We also investigated cytokine stimulation in treated WT*_RAGE_* and SNP*_RAGE_* PBMCs (Figure 2*C*). Cells isolated from SNP*_RAGE_* individuals showed increased expression of *IL17A*, and to a lesser extent, of *IFNG*, as well as decreased expression of *IL4*, in response to the micromolar concentration of S100B and to HMGB1 compared to WT*_RAGE_* cells. No significant differences between genotypes were observed for *IL10*, *IL6* or *TNF* (Figure S2). Stimulation with *A. fumigatus* conidia did not trigger significant *IL17A* expression, a finding consistent with the fact that human anti-*Aspergillus* immunity relies more on Th1 than Th17 type of response [5], [31]. Confirming the human *in vitro* data, a similar inflammatory pattern of cytokine expression was also observed in the lungs of mice infected with *A. fumigatus* and treated with the RAGE ligands (Figure S3; Methods S1).

**Figure 3:**
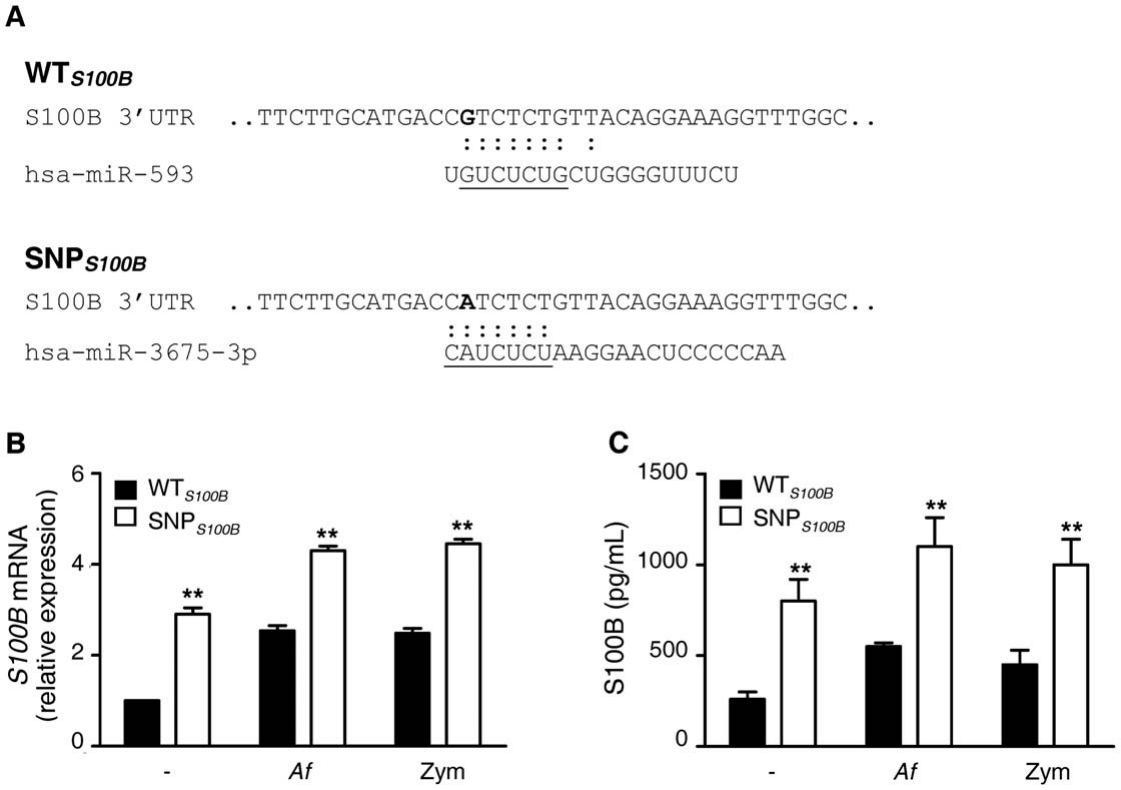
Functional consequences of *S100B* +427C/T polymorphism. (*A*) *In silico* search for miRNAs targeting *S100B* in the vicinity of the polymorphism. Putative binding sites for miR-593 in WT*_S100B_* and miR3675-3p in SNP*_S100B_* sequences are shown. Underlined sequences indicate ‘‘seed” sequences. (*B*) Analysis of *S100B* mRNA expression or (*C*) protein secretion in WT*_S100B_* (wild-type, CC genotype) or SNP*_S100B_* (CT genotype) PBMCs either left untreated or stimulated with *A. fumigatus* conidia (*Af*) and Zymosan (Zym). Data are shown as mRNA relative expression using untreated WT*_S100B_* cells as reference or as absolute quantity of S100B in culture supernatants (for every assay, shown is the mean±SD of data obtained for 10 individuals of each genotype; **P≤0.01 by unpaired *t*-test). Cells from the same individuals were used across conditions in each assay.

**Figure 4:**
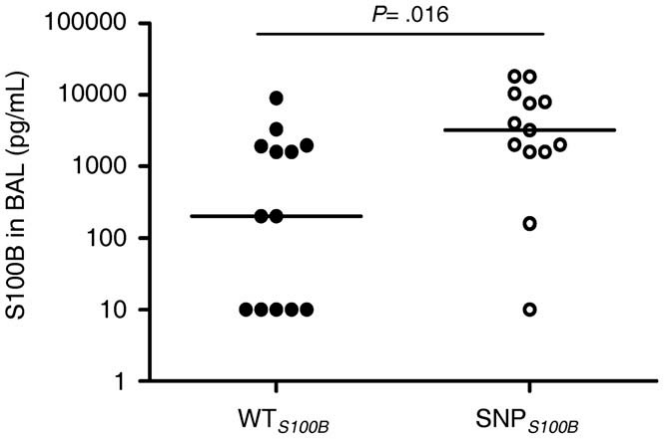
S100B concentrations in BAL samples from patients with proven/probable IA (n = 26) that received graft from wild-type (WT*_S100B_*, n = 13) or polymorphic (SNP*_S100B_*, n = 13) donors. Median S100B levels: 203 pg/mL and 3218 pg/mL for patients with IA receiving graft from WT*_S100B_* or SNP*_S100B_* donors, respectively (*P* = 0.016, by Mann-Whitney *U* test).

To dissect possible molecular mechanisms underlying the effect of the +427C/T polymorphism in *S100B* and given its location in the gene, we examined possible consequences on the binding of regulatory miRNAs. As shown in Figure 3*A*, *in silico* analyses identified alterations in binding sites for miR-593 and miR-3675-3p in the 3′-untranslated sequence of *S100B* that depended on the presence of the polymorphism. Therefore, to investigate whether S100B expression and secretion was modulated by the +427C/T polymorphism, we analyzed PBMCs isolated from WT*_S100B_* (CC genotype) and SNP*_S100B_* (CT genotype) individuals. SNP*_S100B_* PBMCs displayed increased levels of *S100B* mRNA even in basal conditions when compared with WT*_S100B_* cells (Figures 3*B*). Stimulation of cells with *A. fumigatus* conidia or Zym, used as positive control (13), enhanced *S100B* gene induction as compared to the untreated conditions. Similar results were obtained measuring S100B levels in PBMC supernatants where S100B was found to be secreted in higher amounts by polymorphic PBMCs compared to WT*_S100B_* cells (Figure 3*C*). Furthermore, we collected BAL samples from 26 wild-type patients for *S100B* diagnosed with proven/probable IA, 13 of which had *S100B* polymorphic donors and the remaining, wild-type donors. As seen in Figure 4, the median concentration of S100B in BAL samples was significantly higher in patients with IA with a polymorphic donor for *S100B* than in those with a wild-type donor (3218 *vs*. 203 pg/mL; interquartile ranges, 1940–9187 and 10–1632 pg/mL, respectively; *P* = 0.016). Of interest, the levels of sRAGE in BAL samples were not significantly different among patients with distinct patient/donor *RAGE* genotypes (Figure S4; Methods S1).

## Discussion

The immune system has evolved to respond not only to pathogens but also to signals released from damaged and dying cells. The DAMP/RAGE axis is central in danger signaling responses, promoting inflammation and alerting the immune system to the presence of damaging insults. However, sustained activation of danger signaling has been widely implicated in the pathogenesis of multiple diseases with an inflammatory/immune component [14]. Accordingly, several polymorphisms in genes involved in the DAMP/RAGE axis have also been described to influence susceptibility to a number of inflammatory conditions [21], [32]–[36].

In this study, we demonstrate that genetic variants in *RAGE* and *S100B* genes increase susceptibility to IA in HSCT recipients, a finding consistent with recent evidence pointing to a previously unsuspected role of S100B/RAGE-mediated mechanisms in host defense against *A. fumigatus* infection [15]. The specific effect of each genetic variant was found to depend on either patient or donor (*RAGE* -374T/A) or exclusively on donor (*S100B* +427C/T) genetic make-up. This suggests that impairment of RAGE's activity in either patients or donors predisposes to IA, whereas the contribution of S100B-dependent mechanisms relies on its function in the hematopoietic compartment. The fact that these associations were restricted to IA highlights how *A. fumigatus*, more than CMV, is endowed with the ability to trigger danger sensing signaling in the lung, known to express high levels of RAGE under normal conditions [37].

The *RAGE* -374T/A polymorphism has been demonstrated to enhance transcriptional activity by restricting the binding of repressor elements to the gene promoter [19]–[20]. We found increased expression of RAGE in human polymorphic mononuclear cells. RAGE expression was further enhanced upon engagement by its cognate ligands, a finding consistent with the ability of agonists of membrane RAGE to up-regulate its own expression and activate a proinflammatory signaling cascade [20]. Indeed, RAGE activation associated with enhanced expression of proinflammatory cytokines, such as IL-17A and IFN-γ, in human polymorphic cells. Results obtained *in vivo* confirmed that IL-17A was induced in the lungs of mice treated with high concentrations of S100B. Thus, proinflammatory cytokines, known to contribute to progression and severity of *A. fumigatus* infection [38], are activated upon sustained RAGE engagement *in vivo*. Indeed, increased fungal growth and lung inflammation were observed in infected mice treated with high concentrations of S100B [15]. Of interest, the ability of *A. fumigatus* conidia to up-regulate RAGE expression suggests that the fungus itself may be able to induce sustained hyperactivation of the receptor promoting a feed-forward loop towards uncontrolled inflammation that may ultimately favor disease progression.

The +427C/T polymorphism in *S100B* has recently been described to underlie increased serum levels of the protein [21]. Given the location of the polymorphism in the 3′-untranslated region of *S100B*, *in silico* prediction revealed changes in binding sites for the miR-593 and miR3675-3p in the vicinity of the *S100B* polymorphism. Although the functional role of the latter has not yet been defined, miR-593 has been demonstrated to decrease luciferase activity by degrading the mRNA of the reporter gene [39]. Accordingly, we found enhanced expression and secretion of S100B by polymorphic PBMCs in which miR-593 binding is lost. Importantly, high concentrations of this danger molecule were found in BAL samples from HSCT recipients with IA receiving grafts from polymorphic, but not wild-type, donors. This finding, together with the lack of genetic association of the patient polymorphism with IA, indicates that the functional consequence of this polymorphism is contingent upon the hematopoietic compartment, likely myeloid dendritic cells [40], of the donor.

Soluble RAGE (sRAGE), an isoform of RAGE lacking transmembrane and cytosolic domains, acts as a decoy receptor for RAGE ligands in the extracellular compartment, and is believed to afford protection against inflammation and cell injury [41]. Reportedly, sRAGE levels are reduced in patients with inflammatory diseases as compared with healthy subjects [42]. However, the fact that no significant differences in the levels of sRAGE were observed among patients with distinct patient/donor *RAGE* genotypes suggests that neither the increased expression of RAGE nor of S100B was associated with decreased production and functional activity of sRAGE.

In summary, our findings highlight a previously undisclosed role for genetic variants in the S100B/RAGE axis as risk factors to IA in the HSCT setting. Thus, screening of patients for *RAGE* and *S100B* status before HSCT might be useful to a more risk-adapted, prophylactic approach and, whenever possible, for adequate donor selection. The significance of *RAGE* and *S100B* variants should nonetheless be interpreted with caution. Although associations were corrected for known important clinical factors, there are variables in transplanted patients that are difficult to account for. The type of transplant (T-cell depleted) and the size of the cohort may limit the clinical translation of the findings. Despite the fact that validation studies are still currently underway in additional patient cohorts and the mechanisms underlying the increased susceptibility to infection cannot be definitely pinpointed, a genetically-determined hyperfunction of the S100B/RAGE axis is probably involved. Moreover, within the cross-talk between RAGE and TLRs in inflammatory and immune responses with [15] and without infection [43]–[44], it is conceivable that the role of DAMP signaling may go beyond the infection control to include important inflammatory post-transplant events. Furthermore, the finding that miRNAs could target DAMP genes in infection is of potential interest and warrants further investigation. Because expression of miRNAs is unbalanced in various pathological states and, importantly, they are abundantly present and easy detectable in body fluids [45], it is conceivable that miRNAs may serve as novel biomarkers for fungal diseases in HSCT.

## Supporting Information

Figure S1 **Polymorphisms in the S100B/RAGE axis and risk of CMV disease in HSCT recipients.** (*A*) Cumulative incidence of CMV disease according to *RAGE*-374T/A genotype (WT*_RAGE_*, TT; SNP*_RAGE_*, TA+AA). (*B*) Cumulative incidence of CMV disease according to *S100B* +427C/T genotype (WT*_S100B_*, CC; SNP*_S100B_*, CT+TT). From left to right, patients (R) and donors (D).
(TIF)

Figure S2 **Cytokine gene expression in human PBMCs isolated from WT** ***_RAGE_*** **or SNP** ***_RAGE_*** **individuals.** Data are shown as mRNA relative expression of *IL10*, *IL6* and *TNF* using untreated WT*_RAGE_* cells as reference (mean±SD of 10 independent experiments; *P≤0.05 and **P≤0.01 by unpaired *t*-test).
(TIF)

Figure S3 **Cytokine gene expression in the lungs of** ***A. fumigatus*** **-infected mice. **C57BL/6 and *Rage^-/-^* mice were infected with *A. fumigatus* conidia intranasally and were either left untreated (-) or treated with S100B and HMGB1. mRNA levels of *Il17a*, *Ifng*, *Il10* and *Il4* were assessed at 3 days postinfection (n = 6−8 mice from each genotype, 3 independent experiments performed in duplicate; **P≤0.01 by ANOVA).
(TIF)

Figure S4 **Soluble RAGE concentrations in BAL samples from patients with proven/probable IA according to donor and patient genotype (n = 5 for each category).**
(TIF)

Table S1 **Primers used in this study.**
(DOC)

Table S2 **Genotype distributions of** ***RAGE*** **and** ***S100B*** **polymorphisms in hematological patients undergoing HSCT and healthy controls.**
(DOC)

Table S3 **Genotype distribution of** ***RAGE*** **and** ***S100B*** **polymorphisms according to antifungal prophylaxis.**
(DOC)

Methods S1 **Fungal strains, infections, and treatments.** Female C57BL/6, 8 to 10 weeks old mice, were purchased from Charles River Laboratories (Calco, Italy). Homozygous Rage-/- mice were a kind gift from Angelika Bierhaus (Heidelberg, Germany). Mice were bred under specific pathogen-free conditions at the Animal Facility of Perugia University, Perugia, Italy and experiments were performed according to the Italian Approved Animal Welfare Assurance A–3143–01 and the legislative decree 157/2008-B regarding the animal license obtained by the Italian Ministry of Health (2008–2011). All efforts were made to minimize suffering. Viable conidia (> 95%) from the A. fumigatus Af293 strain were obtained by growth on Sabouraud dextrose agar (Difco Laboratories, Detroit) supplemented with chloramphenicol for 4 days at room temperature. Fungi were suspended in endotoxin-free (Detoxi-gel; Pierce Chemical, Rockford, IL, USA) solutions (<1.0 EU/mL, as determined by the Limulus amebocyte lysate method). For infection, mice were anesthetized by intraperitoneal injection (i.p.) of 2.5% avertin (Sigma-Aldrich Co) before instillation of a suspension of 2×107 conidia/20 µL saline intranasally. Mice were treated daily i.p. for 3 consecutive days starting the day of infection with 50 and 500 ng/Kg of purified S100B, 5 and 50 µg/Kg HMGB1 (Sigma-Aldrich Co), at the end of which total RNA was extracted from the lungs. **ELISA for sRAGE**. ELISA was performed using the human RAGE Quantikine assay (R&D Systems, MN, USA) according to the manufacturer's instructions. This assay measures the total pool of sRAGE which is generated either by splicing or cleavage (e.g. sRAGE or esRAGE).
(DOC)
