# Supplementary figures and images for: Genetically-Determined Hyperfunction of the S100B/RAGE Axis Is a Risk Factor for Aspergillosis in Stem Cell Transplant Recipients
Source: PLoS One. 2011 Nov 17;6(11):e27962. doi: 10.1371/journal.pone.0027962 (PMC3219695; doi:10.1371/journal.pone.0027962)

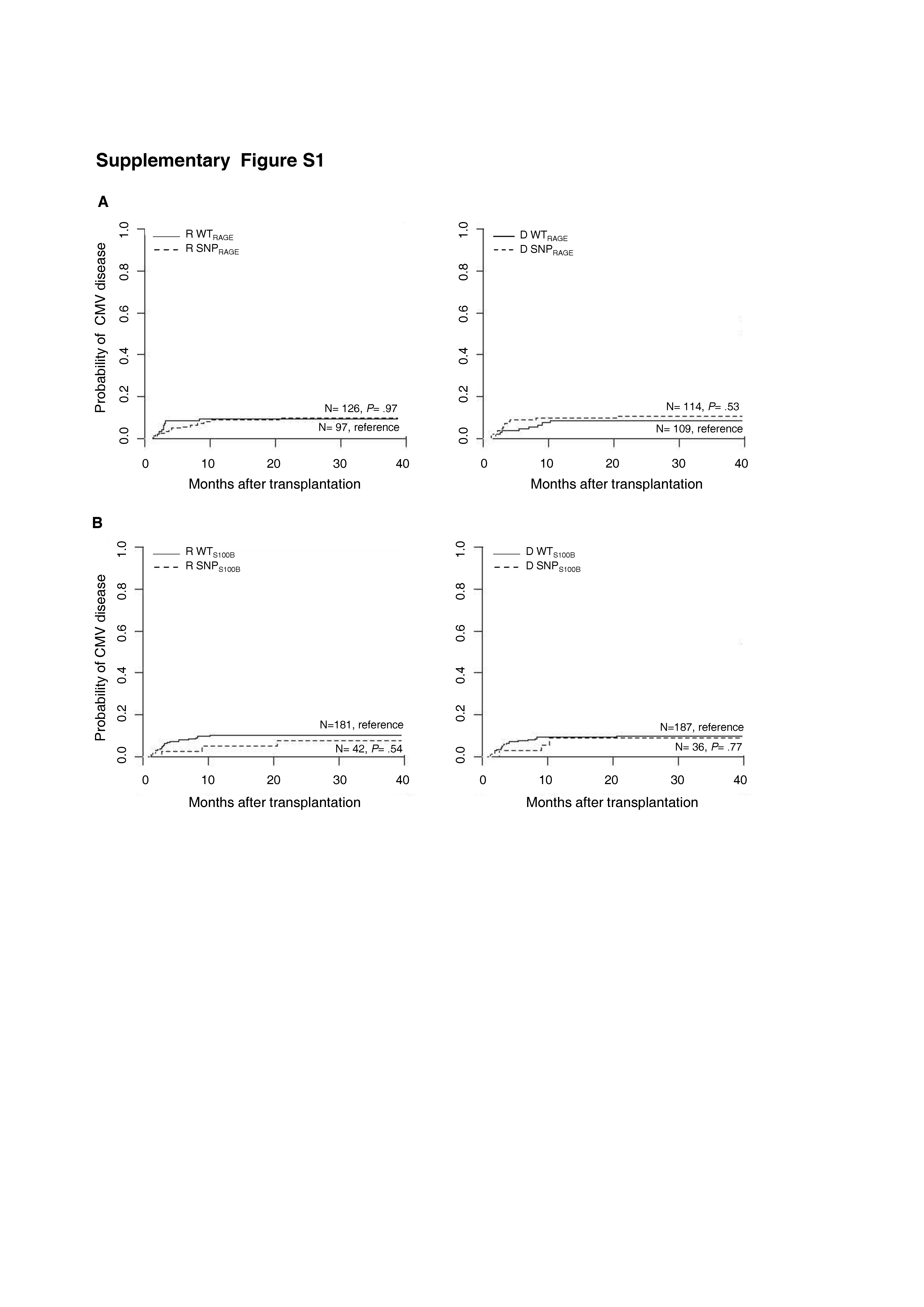

Supplement: Figure S1 — Polymorphisms in the S100B/RAGE axis and risk of CMV disease in HSCT recipients. (A) Cumulative incidence of CMV disease according to RAGE-374T/A genotype (WTRAGE, TT; SNPRAGE, TA+AA). (B) Cumulative incidence of CMV disease according to S100B +427C/T genotype (WTS100B, CC; SNPS100B, CT+TT). From left to right, patients (R) and donors (D). (TIF) [file pone.0027962.s001.tif]

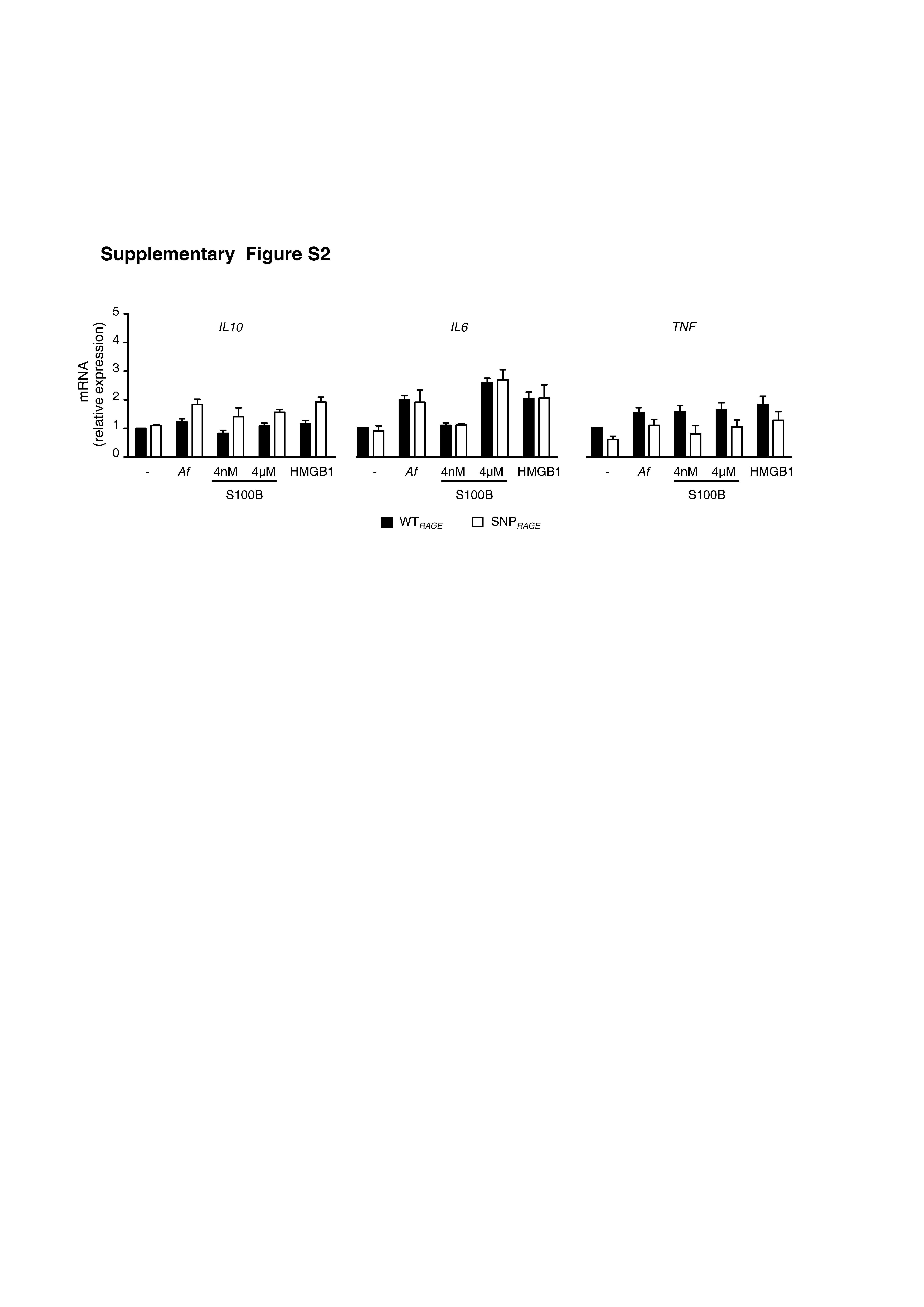

Supplement: Figure S2 — Cytokine gene expression in human PBMCs isolated from WT RAGE or SNP RAGE individuals. Data are shown as mRNA relative expression of IL10, IL6 and TNF using untreated WTRAGE cells as reference (mean±SD of 10 independent experiments; *P≤0.05 and **P≤0.01 by unpaired t-test). (TIF) [file pone.0027962.s002.tif]

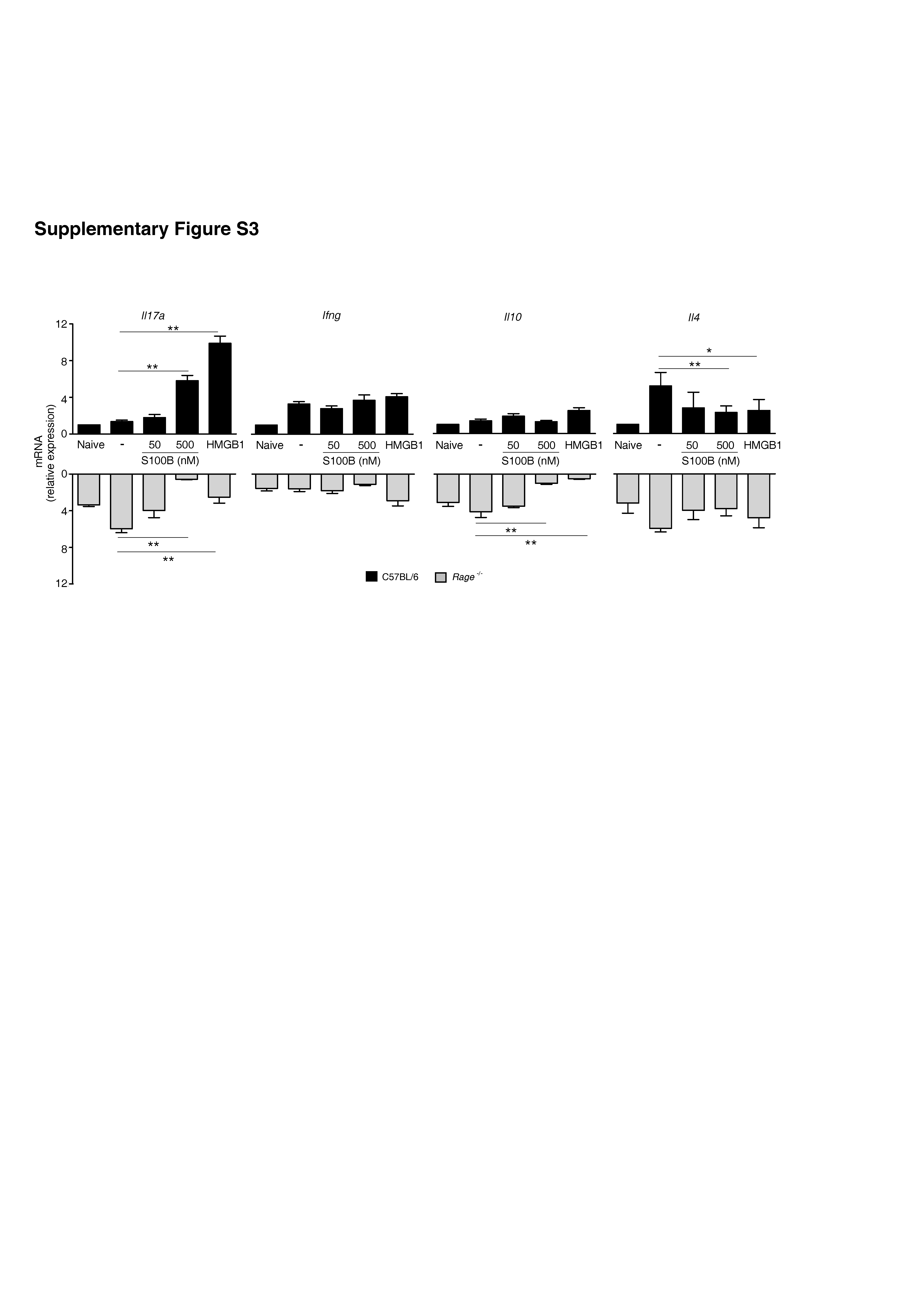

Supplement: Figure S3 — Cytokine gene expression in the lungs of A. fumigatus -infected mice. C57BL/6 and Rage-/- mice were infected with A. fumigatus conidia intranasally and were either left untreated (-) or treated with S100B and HMGB1. mRNA levels of Il17a, Ifng, Il10 and Il4 were assessed at 3 days postinfection (n = 6−8 mice from each genotype, 3 independent experiments performed in duplicate; **P≤0.01 by ANOVA). (TIF) [file pone.0027962.s003.tif]

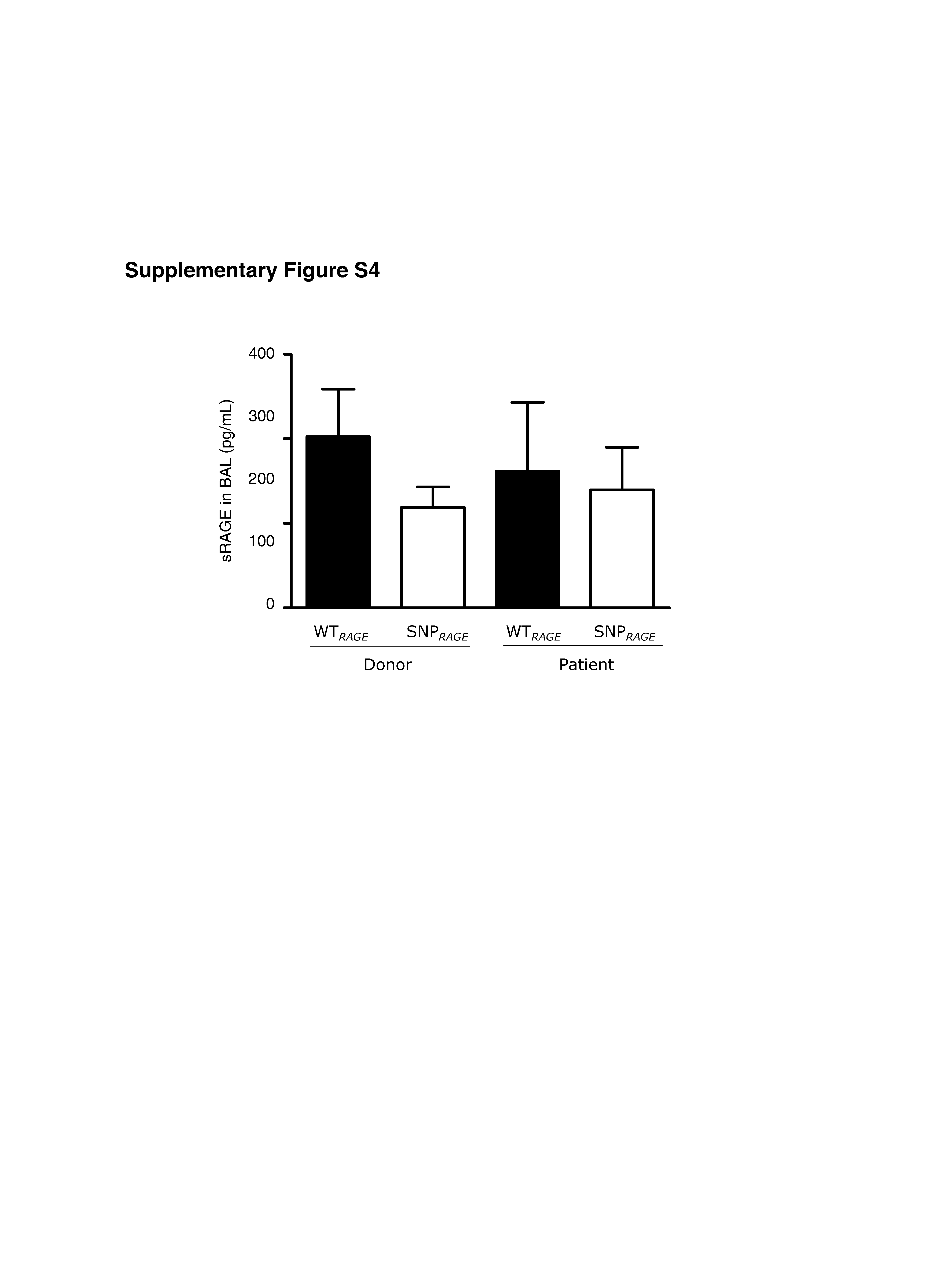

Supplement: Figure S4 — Soluble RAGE concentrations in BAL samples from patients with proven/probable IA according to donor and patient genotype (n = 5 for each category). (TIF) [file pone.0027962.s004.tif]
