# Supplementary material for: Genetically-Determined Hyperfunction of the S100B/RAGE Axis Is a Risk Factor for Aspergillosis in Stem Cell Transplant Recipients
Source: PLoS One. 2011 Nov 17;6(11):e27962. doi: 10.1371/journal.pone.0027962 (PMC3219695; doi:10.1371/journal.pone.0027962)
Supplement: Table S1 — Primers used in this study. (DOC) [file pone.0027962.s005.doc]

**Table S1.** Primers used in this study.

|  | **Forward (5’-3’)** | **Reverse (5’-3’)** |
| --- | --- | --- |
| **Genotyping** |  |  |
| *RAGE* (rs2070600) | CATGATGCAGGCCCAAT | ATTCATGCCTTTGGGACAAG |
|  | CATGATGCAGGCCCAAA |  |
| *RAGE* (rs1800624) | GGCTGGAATGGAAACTGGTA | GGGCCGTTTTCTACTTCTCC |
|  | gggccgggggGTGTCCTTCCCAACA | ggcggcggggAGGAAGAGGGAGCC |
| *S100B* (rs9722) | TATCTGCATGGATGAGGAAC | GTCGGCAACTCCTTTTATCT |
|  | gggccgggggTTCTTGCATGACCA | ggcggcggggTTTCCTGTAACAGAGAC |
| **Real-time RT-PCR** | |  |
| *ACTN* | CACTCTTCCAGCCTTCCTTCC | ACAGCACTGTGTTGGCGTAC |
| *RAGE* | GCCAGAAGGTGGAGCAGTAG | CCAGTGGATTTGAGGAGAGG |
| *S100B* | ATTCTGGAAGGGAGGGAGAC | TCCACAACCTCCTGCTCTTT |
| *IL17A* | ACCACATGAACTCTGTCCCC | CCCACGGACACCAGTATCTT |
| *IFNG* | AGCTCTGCATCGTTTTGGGTT | GTTCCATTATCCGCTACATCTGAA |
| *IL10* | GCCTAACATGCTTCGAGATC | TGATGTCTGGGTCTTGGTTC |
| *IL4* | TGCCTCCAAGAACACAACTG | GTCCTTCTCATGGTGGCTGT |
| *IL6* | AAAGAGGCACTGGCAGAAAA | TTTCACCAGGCAAGTCTCCT |
| *TNF* | AACCTCCTCTCTGCCATCAA | GGAAGACCCCTCCCAGATAG |
