# Supplementary material for: Genetically-Determined Hyperfunction of the S100B/RAGE Axis Is a Risk Factor for Aspergillosis in Stem Cell Transplant Recipients
Source: PLoS One. 2011 Nov 17;6(11):e27962. doi: 10.1371/journal.pone.0027962 (PMC3219695; doi:10.1371/journal.pone.0027962)
Supplement: Table S2 — Genotype distributions of RAGE and S100B polymorphisms in hematological patients undergoing HSCT and healthy controls. (DOC) [file pone.0027962.s006.doc]

**Table S2.** Genotype distributions of *RAGE* and *S100B* polymorphisms in hematological patients undergoing HSCT and healthy controls.

| **Genotype** | **Healthy**  **(n=468)** | **AML**  **(n=108)** | ***P* *** | **ALL**  **(n=46)** | ***P* *** | **HL**  **(n=24)** | ***P* *** | **NHL**  **(n=19)** | ***P* *** | **CLL**  **(n=15)** | ***P* *** | **MM**  **(n=11)** | ***P* *** |
| --- | --- | --- | --- | --- | --- | --- | --- | --- | --- | --- | --- | --- | --- |
| **WT*_RAGE_*** | 215 (45.9%) | 44 (40.7%) |  | 21 (45.7%) |  | 10 (41.7%) |  | 11 (57.9%) |  | 5 (33.3%) |  | 6 (54.5%) |  |
| **SNP*_RAGE_*** | 253 (54.1%) | 64 (59.3%) | 0.34 | 25 (54.3%) | 1.00 | 14 (58.3%) | 0.83 | 8 (42.1%) | 0.35 | 10 (66.7%) | 0.43 | 5 (45.5%) | 0.76 |
| **WT*_S100B_*** | 390 (83.3%) | 91 (84.3%) |  | 38 (82.6%) |  | 17 (66.7%) |  | 17 (89.5%) |  | 11 (73.3%) |  | 9 (81.8%) |  |
| **SNP*_S100B_*** | 78 (16.7%) | 17 (15.7%) | 0.89 | 8 (17.4%) | 1.00 | 7 (32.3%) | 0.16 | 2 (10.5%) | 0.56 | 4 (26.7%) | 0.48 | 2 (18.2%) | 1.00 |

WT – wild-type; SNP – single nucleotide polymorphism. WT*_RAGE_* – TT genotype; SNP*_RAGE_* – TA + AA genotypes; WT*_S100B_* – CC genotype; SNP*_S100B_* – CT + TT genotypes; AML – acute myeloid leukemia; ALL – acute lymphoid leukemia; HL – Hodgkin lymphoma; NHL – non-Hodgkin lymphoma; CLL – chronic lymphocytic leukemia; MM – multiple myeloma.

**P* values are from Fisher’s exact test.
