# Supplementary material for: Genetically-Determined Hyperfunction of the S100B/RAGE Axis Is a Risk Factor for Aspergillosis in Stem Cell Transplant Recipients
Source: PLoS One. 2011 Nov 17;6(11):e27962. doi: 10.1371/journal.pone.0027962 (PMC3219695; doi:10.1371/journal.pone.0027962)
Supplement: Table S3 — Genotype distribution of RAGE and S100B polymorphisms according to antifungal prophylaxis. (DOC) [file pone.0027962.s007.doc]

**Table S3.** Genotype distribution of *RAGE* and *S100B* polymorphisms according to antifungal prophylaxis.

| **Genotype** | **Fluconazole (n=28)** | **LAmB**  **(n=195)** | ***P* *** |
| --- | --- | --- | --- |
| **D WT*_RAGE_*** | 17 (60.7%) | 91 (46.7%) |  |
| **D SNP*_RAGE_*** | 11 (39.3%) | 104 (53.3%) | 0.22 |
| **R WT*_RAGE_*** | 16 (57.1%) | 81 (41.5%) |  |
| **R SNP*_RAGE_*** | 12 (42.9%) | 114 (58.5%) | 0.15 |
| **D WT*_S100B_*** | 22 (78.6%) | 166 (85.1%) |  |
| **D SNP*_S100B_*** | 6 (21.4%) | 29 (14.9%) | 0.40 |
| **R WT*_S100B_*** | 20 (71.4%) | 164 (84.1%) |  |
| **R SNP*_S100B_*** | 8 (28.6%) | 31 (15.9%) | 0.11 |

LAmB – liposomal amphotericin-B; D – donor; R – recipient; WT – wild-type; SNP – single nucleotide polymorphism; WT*_RAGE_* – TT genotype; SNP*_RAGE_* – TA + AA genotypes; WT*_S100B_* – CC genotype; SNP*_S100B_* – CT + TT genotypes.

^*^*P* values are from Fisher’s exact test.
