# Supplementary material for: Genetically-Determined Hyperfunction of the S100B/RAGE Axis Is a Risk Factor for Aspergillosis in Stem Cell Transplant Recipients
Source: PLoS One. 2011 Nov 17;6(11):e27962. doi: 10.1371/journal.pone.0027962 (PMC3219695; doi:10.1371/journal.pone.0027962)
Supplement: Methods S1 — Fungal strains, infections, and treatments. Female C57BL/6, 8 to 10 weeks old mice, were purchased from Charles River Laboratories (Calco, Italy). Homozygous Rage-/- mice were a kind gift from Angelika Bierhaus (Heidelberg, Germany). Mice were bred under specific pathogen-free conditions at the Animal Facility of Perugia University, Perugia, Italy and experiments were performed according to the Italian Approved Animal Welfare Assurance A–3143–01 and the legislative decree 157/2008-B regarding the animal license obtained by the Italian Ministry of Health (2008–2011). All efforts were made to minimize suffering. Viable conidia (> 95%) from the A. fumigatus Af293 strain were obtained by growth on Sabouraud dextrose agar (Difco Laboratories, Detroit) supplemented with chloramphenicol for 4 days at room temperature. Fungi were suspended in endotoxin-free (Detoxi-gel; Pierce Chemical, Rockford, IL, USA) solutions (<1.0 EU/mL, as determined by the Limulus amebocyte lysate method). For infection, mice were anesthetized by intraperitoneal injection (i.p.) of 2.5% avertin (Sigma-Aldrich Co) before instillation of a suspension of 2×10^7^ conidia/20 µL saline intranasally. Mice were treated daily i.p. for 3 consecutive days starting the day of infection with 50 and 500 ng/Kg of purified S100B, 5 and 50 µg/Kg HMGB1 (Sigma-Aldrich Co), at the end of which total RNA was extracted from the lungs. ELISA for sRAGE. ELISA was performed using the human RAGE Quantikine assay (R&D Systems, MN, USA) according to the manufacturer's instructions. This assay measures the total pool of sRAGE which is generated either by splicing or cleavage (e.g. sRAGE or esRAGE). (DOC) [file pone.0027962.s008.doc]

**Methods S1**

**Fungal strains, infections, and treatments.** Female C57BL/6, 8 to 10 weeks old mice, were purchased from Charles River Laboratories (Calco, Italy). Homozygous *Rage*^-/-^ mice were a kind gift from Angelika Bierhaus (Heidelberg, Germany). Mice were bred under specific pathogen-free conditions at the Animal Facility of Perugia University, Perugia, Italy and experiments were performed according to the Italian Approved Animal Welfare Assurance A–3143–01 and the legislative decree 157/2008-B regarding the animal license obtained by the Italian Ministry of Health (2008-2011). All efforts were made to minimize suffering. Viable conidia (> 95%) from the *A. fumigatus* Af293 strain were obtained by growth on Sabouraud dextrose agar (Difco Laboratories, Detroit) supplemented with chloramphenicol for 4 days at room temperature. Fungi were suspended in endotoxin-free (Detoxi-gel; Pierce Chemical, Rockford, IL, USA) solutions (<1.0 EU/mL, as determined by the Limulus amebocyte lysate method). For infection, mice were anesthetized by intraperitoneal injection (i.p.) of 2.5% avertin (Sigma-Aldrich Co) before instillation of a suspension of 2×10^7^ conidia/20 µL saline intranasally. Mice were treated daily i.p. for 3 consecutive days starting the day of infection with 50 and 500 ng/Kg of purified S100B, 5 and 50 μg/Kg HMGB1 (Sigma-Aldrich Co), at the end of which total RNA was extracted from the lungs.

**ELISA for sRAGE**. ELISA was performed using the human RAGE Quantikine assay (R&D Systems, MN, USA) according to the manufacturer’s instructions. This assay measures the total pool of sRAGE which is generated either by splicing or cleavage (e.g. sRAGE or esRAGE).
